# Effect of Different Drying Methods and Pretreatments on Cyanogenic Glycosides, Calcium Oxalate, and Bioactive Compounds of *Arum rupicola* Boiss. var. *virescens* (Stapf) P.C. Boyce

**DOI:** 10.3390/plants15203241

**Published:** 2026-10-21

**Authors:** Serdar Uğurlu, Emre Bakkalbaşı

**Affiliations:** 1 Department of Plant and Animal Production, Yüksekova Vocational School, Hakkari University, Hakkari 30100, Türkiye; 2 Department of Food Engineering, Faculty of Engineering, Van Yüzüncü Yıl University, Zeve Campus, Van 65080, Türkiye; emrebakkalbasi@yyu.edu.tr

**Keywords:** drying, pretreatment, cyanogenic glycoside, calcium oxalate

## Abstract

In this study, the effects of vacuum combined infrared radiation (VCIR), microwave (MW), conventional (CV), shade, and sun drying of *Arum rupicola* Boiss. var. *virescens* (Stapf) P.C. Boyce leaves were investigated for their effects on the contents of certain compounds, including cyanogenic glycosides and calcium oxalate. Additionally, the effects of ultrasonic (100% amplitude, 10, 20, and 30 min), blanching (90 °C for 5 and 10 min), and freeze-thawing (−18 °C for 16 h) pretreatments on the cyanogenic glycoside and calcium oxalate contents in the samples were also investigated. The drying methods affected the total phenolic, total flavonoid, total chlorophyll, and total carotenoid contents. In addition, the antioxidant activity of these compounds was assessed, with higher activity observed in samples dried using VCIR. The cyanogenic glycoside amount and calcium oxalate raphide crystal sizes decreased compared to the fresh sample. The most effective method for reducing the cyanogenic glycoside content and the raphide crystal size is VCIR among the drying processes and 30 min of ultrasonication among the pretreatments.

## 1. Introduction

The Araceae, known as the Arum family, are a family of monocotyledonous flowering plants [1]. Arum is native to Europe, northern Africa, and western and central Asia. It is also found in the Mediterranean region with the highest species diversity [2]. Arum plants contain members of several chemical groups such as alkaloids, polyphenols, glycosides (flavonoids, saponins, and cyanogenic glycosides), monoterpenes, sesquiterpenes, and lectins [3]. Extracts from Arum species possess analgesic, antimicrobial, anti-inflammatory, and anticancer effects [3]. Despite some positive health effects, members of the genus Arum are also associated with some toxic effects. The toxicity of Arum subspecies is caused by several individual compounds or chemical groups. Oxalate crystals and cyanogenic glycosides, along with other components such as volatile amines and alkaloids, are the main causes of the plant’s toxicity [4,5]. To avoid the poisonous effects of *Arum rupicola*, its leaves and rhizomes are cooked with dried mulberries, sumac, pomegranate sauce, and ground wheat [6].

Drying is one of the methods used to reduce or inactivate oxalate in different foods. Drying is one of the most energy-intensive processes in postharvest processing. It is used to reduce the water content of products such as fruit, vegetables, herbs, and other agricultural produce after harvest. Reducing the water content prolongs the shelf-life of bio-products by lowering the water activity to a level where microorganisms, enzymes, and other reactions are stopped. Plants are also dried for effective extraction of the active ingredients or to reduce the toxic effects [7]. Wanyo et al. [8] reported that the total, soluble, and insoluble oxalate contents of sample were generally reduced by various drying processes. Furthermore, pretreatment methods can also be employed to reduce or eliminate the oxalate content of food. Boiling generally lead to a reduction in the total, soluble, and insoluble oxalate contents [8]. Consuming foods containing toxic cyanogens can also result in acute or chronic toxicity, which can lead to high mortality and morbidity rates. However, the hydrocyanic content can be significantly reduced by applying various processing methods, thereby decreasing the potential health risk [9]. Drying is one of the most effective methods of removing cyanogenic glycosides from food plants. Several drying methods can be used to reduce the amount of cyanogens in food products [10]. Microwave heating, hydrothermal treatment, and their combination have been reported to cause a significant reduction in the cyanogenic glycosides of plum kernels [11].

*Arum rupicola* Boiss. var. *virescens* (Stapf) P.C. Boyce is a plant with a broad tuberous underground portion and a scape 20–60 cm long. The leaves are heart-shaped. The spathe is greenish in color on both the outer and inner surfaces. The spadix is 9–33 cm long, with a yellowish-brown tip. Its habitats include wooded areas, rocky and stony slopes, and roadside edges in Eastern Anatolia. The leaves are boiled, drained, and added to pilaf [12]. Therefore, this study aims to determine the effects of different drying methods (VCIR, MW, CV, shade, and sun) and pretreatment applications (ultrasound, blanching, freeze-thawing) on the bioactive and toxic components of *Arum rupicola* Boiss. var. *virescens* (Stapf) P.C. Boyce leaves.

## 2. Results and Discussion

### 2.1. Effect of Drying on Some Quality Parameters of Samples

The initial moisture content of fresh *Arum rupicola* Boiss. var. *virescens* (Stapf) P.C. Boyce leaves was 88.09%. The final moisture content of the dried products was lower than 9.52%. Among the methods (Table 1), microwave drying is the fastest technique, requiring only 10 min at a power level of 300 W. These results are similar to those obtained in drying studies of *Paris polyphylla* Smith [13]. The drying rate required to achieve a fixed moisture content varies depending on the drying method: MW > VCIR > CV > sun > shade drying. The reason for these differences is that higher temperatures and greater penetration capability result in shorter drying times [13]. Drying times with MW were found to be 5 and 18 times shorter than those with VCIR and CV methods, respectively. Furthermore, the VCIR method provided a drying time approximately 3.6 times shorter than that of CV. Generally, the new drying techniques offer shorter processing times compared to conventional methods. The times for MW, VCIR, and CV dryers were found to be significantly lower compared to drying in the sun and shade. Therefore, it was concluded that artificial dryers provide faster and more efficient drying compared to natural drying methods. Although the current findings demonstrate the technical efficiency of these methods, future studies that comparatively evaluate different drying techniques in terms of energy costs and economic sustainability parameters will make a significant contribution to the literature.

The effects of different drying methods on the TPC, TFC, and antioxidant activity (ABTS and DPPH) are shown in Table 1. The TPC, TFC, ABTS, and DPPH values were significantly affected by the drying methods. Compared to the fresh sample, significant increases in the TPC, TFC, ABTS, and DPPH values were observed in samples dried using the VCIR and MW methods (*p* < 0.05). This may be due to the release of numerous free phenolic compounds as a result of the disruption of the structure of the sample cells [14]. The highest results were attained with VCIR drying. When compared with MW, VCIR drying took a longer time. It can be assumed that the lower watt and vacuum application in VCIR provide protection against the oxidation of phenolics. In contrast, significant decreases in these parameters were detected in samples dried in the shade and sun (*p* < 0.05). This may be due to the samples being exposed to more oxidation during the longer drying period. The lowest TPC, TFC, ABTS, and DPPH were observed in sun-dried samples. The change in the TFC was similar to that of the TPC. Similar findings were observed in a study of *Codonopsis pilosulae* slices by Yue et al. [14].

The total chlorophyll and carotenoid contents in the fresh sample were 3599.02 mg/kg DM and 965.43 mg β-car.eq./kg DM, respectively. Alaca et al. [15] found lower levels of these compounds (1435.16 mg/kg DM and 572.83 mg β-carotene equivalents/kg DM) in Arum *conophalloides* Kotschy ex Schott var. *conophalloides*. It is believed that this difference was caused by the use of different species. Table 1 shows that various drying conditions resulted in significant differences in the total chlorophyll and carotenoid contents of the samples (*p* < 0.05). The highest total chlorophyll and carotenoid content were detected in the sample dried using the VCIR method; this was followed by samples dried using the MW, shade, CV, and sun methods, and statistically significant differences (*p* < 0.05) were observed among them. In general, it was observed that longer drying times or high temperatures resulted in greater degradation of total chlorophyll and carotenoids in the dried sample. The collapse of plant tissues during the drying process could lead to the release of chlorophyll molecules from the protein complex. This could promote the transformation of chlorophyll into pheophytin due to long-term heat exposure. This could also result in the release of substrates for enzymatic browning reactions into the surrounding areas [16]. Among all conditions, CV and sun drying caused the greatest losses in total chlorophylls and carotenoids of samples. Alibas et al. [17] reported that chlorophyll values in *Ocimum basilicum* L. leaves decreased significantly during natural, convective, and microwave drying. VCIR preserves carotenoid compounds more effectively than other drying methods. This behavior is related to the lack of oxygen required for oxidation reactions. Carotenoids contain numerous double bonds that are sensitive to oxidation occurring at high temperatures and/or in the presence of air in the environment [18].

The chlorogenic and caffeic acids in the samples were determined as individual phenolics. Alaca et al. [15] detected chlorogenic acid in a sample of *Arum conophalloides* Kotschy ex Schott var. *conophalloides* and found that its concentration was higher than the value reported in our study. As noted earlier, this may be due to species differences. The amount of these phenolics in the dried samples ranged from 81.16 to 301.75 and 49.62 to 101.06 mg/kg DM, respectively, while those of the fresh sample were 136.87 and 170.78 mg/kg DM, respectively. The effect of drying techniques on the phenolic compounds in the sample was found to be significant (*p* < 0.05). Compared to the fresh sample, caffeic acid decreased with drying. A similar situation for caffeic acid has also been observed in apricot samples dried using different drying methods (hot air, microwave, and hot air–microwave combination) [19]. Chlorogenic acid is sensitive to oxygen and heat, which makes it susceptible to degradation in high-temperature and atmospheric conditions [20]. However, our results showed that while chlorogenic acid decreased in the VCIR and CV samples, it increased in the MW, shade, and sun-dried samples. Our results also show that drying the samples using different drying methods profoundly affected the qualitative phenolic composition, increasing the content of some compounds while decreasing the content of others.

Ascorbic acid, as a strong antioxidant, is very unstable and sensitive to light, heat, and oxygen, so it is mainly used as an indicator to assess different processes [21]. Fresh *Arum rupicola* Boiss. var. *virescens* (Stapf) P.C. Boyce leaves are rich in L-ascorbic acid (16,209.19 ± 627.04 mg/kg DM) (Table 1). A similar situation has been reported in the species Arum *conophalloides* Kotschy ex Schott var. *conophalloides* (10,589.7 ± 850.2 mg/kg DM) [15]. All drying methods significantly reduced the ascorbic acid content of *Arum rupicola* Boiss. var. *virescens* (Stapf) P.C. Boyce leaves compared to fresh leaves; decreases of 16.17% in VCIR, 17.29% in MW, 23.46% in CV, 82.67% in shade, and 84.75% in sun were observed. While the lowest ascorbic acid loss was determined with VCIR, the highest losses were detected in the sun and shade samples, which had long drying times. High amounts of L-ascorbic acid (36–55%) loss were also detected in nettle samples dried using different drying methods (freeze-drying, oven-drying, heat-pump drying) [22].

HMF formation is widely used in the assessment of food quality and as an indicator of excessive heating and thermal damage [23]. All dried samples had low HMF contents (2.47–17.45) (Table 1). However, the drying methods significantly affected the HMF content of the samples (*p* < 0.05). The results showed that very low amounts of HMF were observed in VCIR samples, while the highest HMF contents were detected in MW and sun-dried samples. Demircan et al. dried samples using hot air, sun, oven, freeze, infrared, infrared-assisted microwave, and microwave drying. The highest HMF content was reported in samples dried using a microwave [24]. The significant increase in HMF during MW drying may be related to the primary step of the Maillard reaction, which promotes HMF formation in the samples [25]. The HMF content in our study was considered very low compared to the HMF contents previously reported in roasted coffee (300–2900 mg/kg) and dried fruits such as pineapple (280 mg/kg), pear (100 mg/kg), and plum (1600–2200 mg/kg) [26]. No toxic effects of HMF have been observed at daily doses ranging from 80 to 100 mg/kg body weight [27]. The HMF levels in all *Arum rupicola* Boiss. var. *virescens* (Stapf) P.C. Boyce leaf samples prepared in this study would not be harmful to health.

### 2.2. Cyanogenic Glycoside Content, Calcium Oxalate Raphide Crystals and Effects of Different Pretreatments

**Figure 1:**
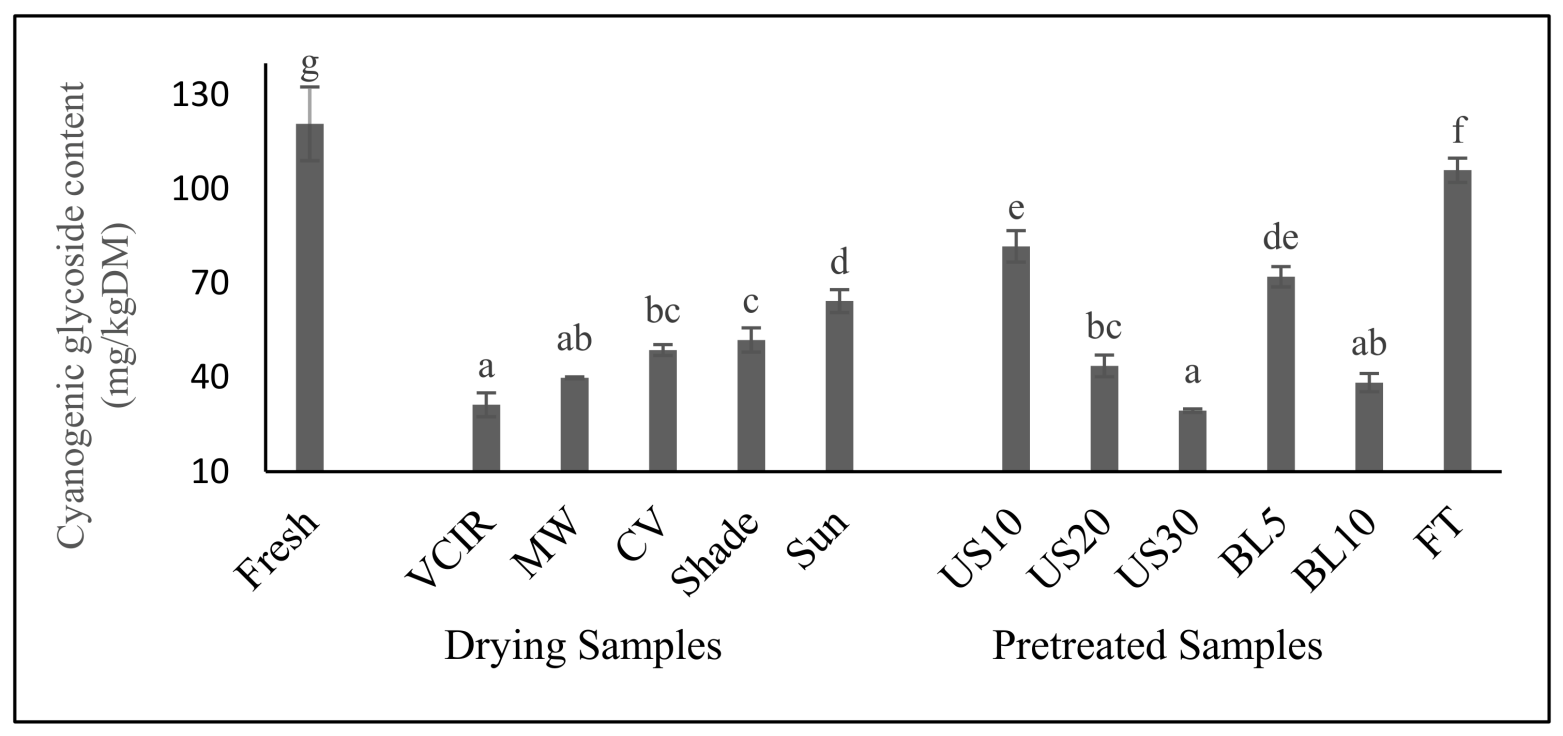
Cyanogenic glycoside content of *Arum rupicola* Boiss. var. *virescens* (Stapf) P.C. Boyce leaves. Data are expressed as mean ± SD. Different lowercase letters indicate significant differences among the samples. VCIR: Vacuum combined infrared radiation, MW: Microwave drying, CV: Conventional drying, Shade: Shade drying, Sun: Sun drying, US10: Ultrasound treatment for 10 min, US20: Ultrasound treatment for 20 min, US30: Ultrasound treatment for 30 min, BL5: Blanching treatment for 5 min, BL10: Blanching treatment for 10 min, FT: Frozen.

The cyanogenic glycoside content in the fresh samples was found to be 120.72 mg/kg DM. A significant decrease (*p* < 0.05) in the cyanogenic glycoside content was found in the dried samples as compared to the fresh sample (Figure 1). The cyanogenic glycoside content was significantly reduced by 74.08%, 66.94%, 59.65%, 56.99%, and 46.73% in the VCIR, MW, CV, shade, and sun samples, respectively. A similar situation has also been reported in bamboo shoot samples dried with different drying methods [28]. Cyanogenic glycosides are water-soluble compounds that are stored in separate compartments in intact cells. The key characteristic of these compounds is the formation of hydrocyanic acid upon tissue disruption by different factors, such as cutting and heating [11]. Specifically, the decrease in dried samples is very rapid due to the heat, causing the vaporization of hydrocyanic acid (a boiling point of 26 °C) released as a result of decomposition [28]. The highest reduction in our study was observed in the VCIR sample. This may be due to the vacuum rapidly removing the HC formed from the environment, in addition to the heat applied in the VCIR. Despite the significant difference in drying time and temperature between CV and shade-dried samples, the reduction rates were similar (*p* > 0.05). The reason for the greater decrease in hydrocyanic acid at lower temperatures may be related to the longer drying time. This allows sufficient time for the hydrocyanic acid to be released into the environment properly [9].

The effect of different pretreatments (ultrasound, blanching, and freeze-thawing) on the amount of cyanogenic glycosides is also given in Figure 1. Cyanogenic glycosides were reduced to 29.44 mg/kg DM with ultrasound compared to the fresh sample (120.72 mg/kg DM). As the ultrasonication time increased, the amount of cyanogenic glycosides decreased significantly (*p* < 0.05). The cyanogenic glycoside content of the sample decreased with increasing sonication time. Sonication treatment for 30 min eliminated 75.61% of the cyanogenic glycosides. Ultrasound pretreatment damages tissue cell walls when the treatment duration is extended [29]. After blanching for 5 and 10 min, 40.32% and 68.24% of the cyanogenic glycosides were removed compared to the fresh sample. The decrease in cyanogenic glycosides after blanching is associated with the hydrolysis of these compounds into hydrocyanic acid and the evaporation of the resulting products [9]. When the effect on cyanogenic glycosides of the FT pretreatment was examined, a 12.23% decrease was observed compared to the fresh sample. A low decrease in cyanogenic glycosides was observed. This indicates that the effect of the FT pretreatment is less pronounced. Compared to fresh samples, all drying and pretreatment methods were found to reduce the amount of cyanogenic glycosides. US30 was found to be the most effective pretreatment in the elimination of cyanogenic glucosides (*p* < 0.05). In addition, similar results in the removal of cyanogenic glycosides between US10 and BL5, and US30 and BL10 pretreatments were observed (*p* > 0.05). US30 pretreatment was found to be more effective than the drying methods in removing cyanogenic glycosides.

**Figure 2:**
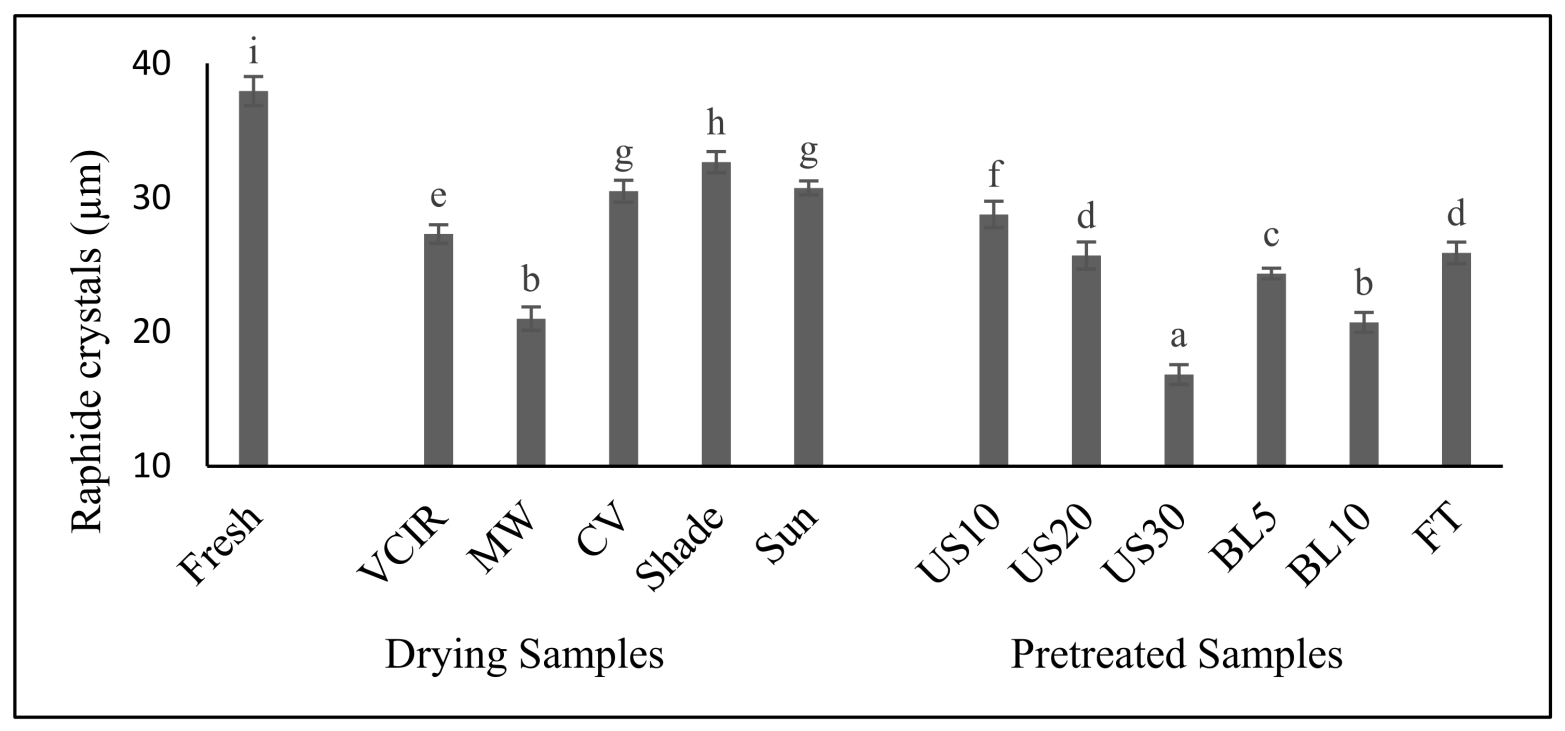
Calcium oxalate raphide crystals of *Arum rupicola* Boiss. var. *virescens* (Stapf) P.C. Boyce leaves. Data are expressed as mean ± SD. Different lowercase letters indicate significant differences among the samples. VCIR: Vacuum combined infrared radiation, MW: Microwave drying, CV: Conventional drying, Shade: Shade drying, Sun: Sun drying, US10: Ultrasound treatment for 10 min, US20: Ultrasound treatment for 20 min, US30: Ultrasound treatment for 30 min, BL5: Blanching treatment for 5 min, BL10: Blanching treatment for 10 min, FT: Frozen.

**Figure 3:**
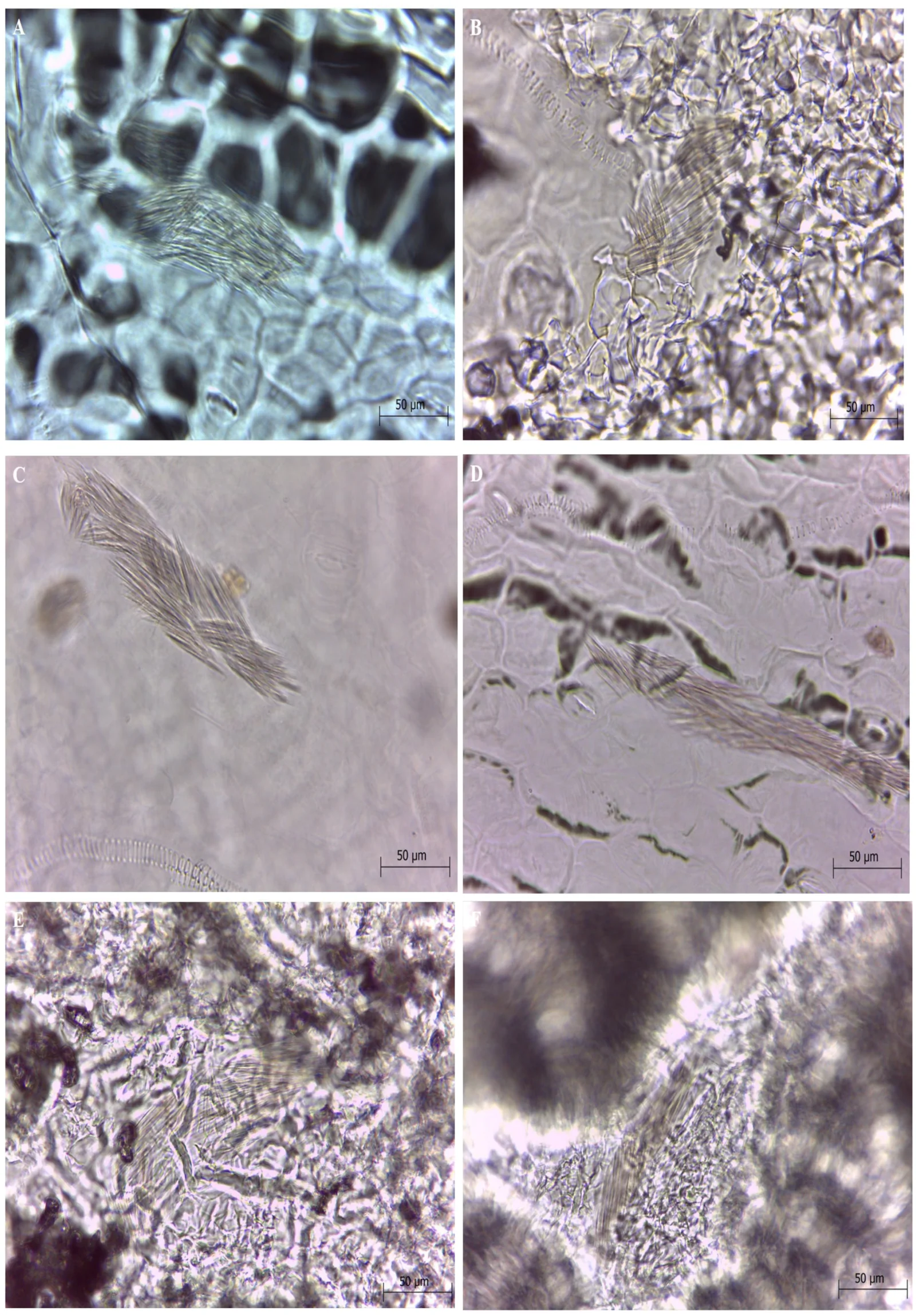
Calcium oxalate raphide crystals in samples dried using different drying methods ((**A**): Fresh, (**B**): Vacuum combined infrared radiation, (**C**): Microwave, (**D**): Conventional, (**E**): Shade, (**F**): Sun).

**Figure 4:**
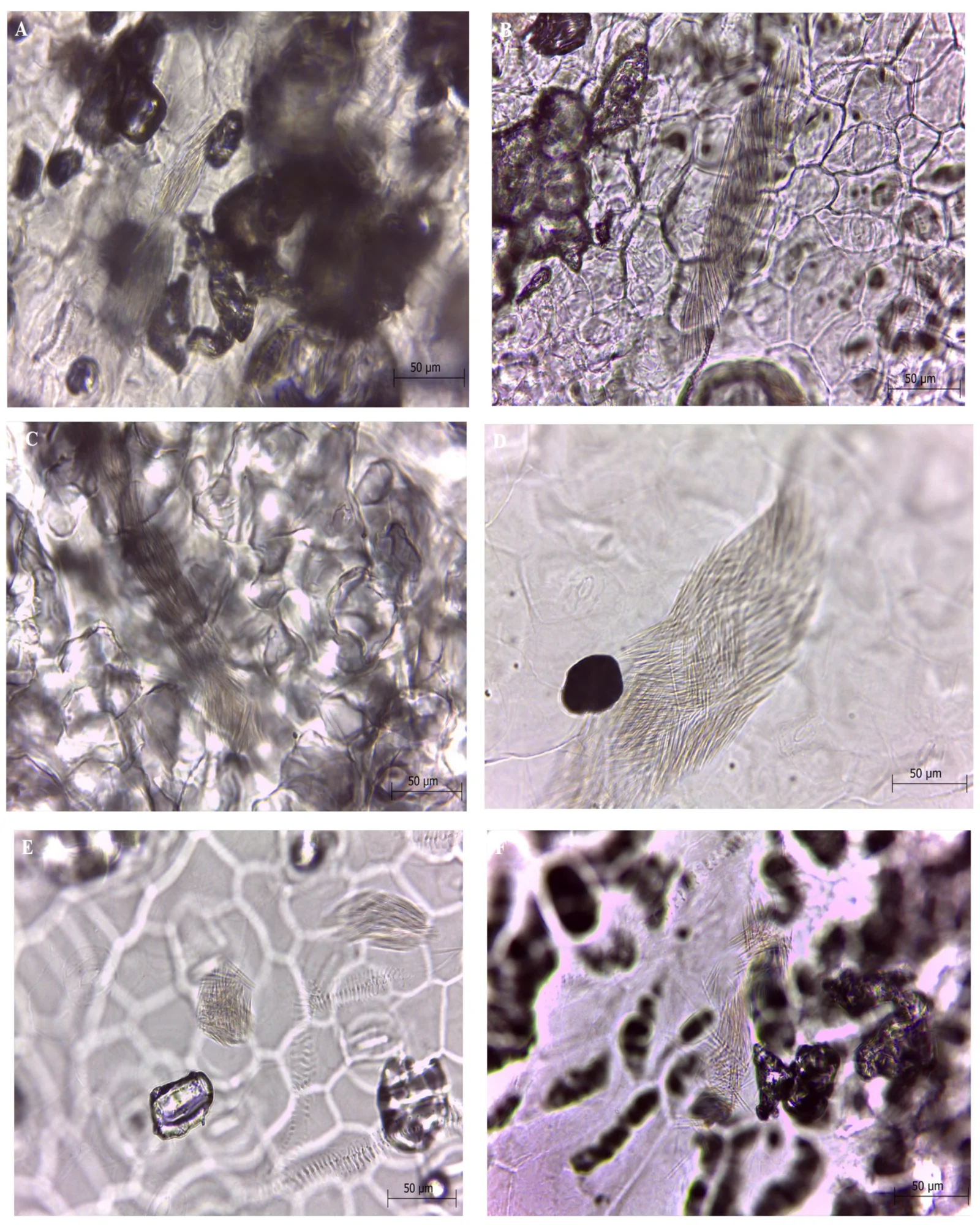
Calcium oxalate raphide crystals in samples subjected to different pretreatments ((**A**): US10, (**B**): US20, (**C**): US30, (**D**): BL5, (**E**): BL10, (**F**): FT).

This study focused on raphide crystals, which are calcium oxalate crystals. The effect of different drying methods and pretreatments on the length of calcium oxalate crystals of Arum is shown in Figure 2. The morphology of calcium oxalate crystals in the samples is presented in Figure 3 and Figure 4. In our study, raphide crystals appeared as bundles. Raphide crystals in *Amorphophallus muelleri* Blume were found to exhibit single and cluster crystal forms [30]. The length of the raphide crystals in the fresh sample was found to be 37.93 ± 1.08 µm. In a similar study examining the characteristics of ten genera belonging to the Araceae family, raphide crystals appeared as spindle-shaped bundles, and the lengths of the crystals ranged from 47.4 μm to 175.07 μm [31]. The reported values were found to be higher than the values in our study. This situation is thought to be influenced by the different types and cultivation conditions. Compared to the fresh sample, the length of the raphide crystals decreased by 13.94–55.65% in all samples (*p* < 0.05). In the dried samples, the lengths of the raphide crystals varied as follows: MW < VCIR < CV ≤ sun < shade. The samples treated with MW had the smallest size because the acute heat stress formed inside the cells during microwave heating led to structural damage to calcium oxalate [32]. It has been reported that different drying methods (hot air, vacuum, and freeze) significantly reduce the insoluble oxalate content in dried samples compared to fresh samples [8]. Oxalates are known to inhibit calcium absorption by forming insoluble salts with calcium. However, diets rich in foods containing insoluble oxalates are unlikely to be harmful. Insoluble oxalates in vegetables are converted to soluble oxalates as the sample is dried. Oxalate degradation occurs through oxidation, decarboxylation, and acetylation [8]. In our study, the drying process may have reduced the size of calcium oxalates. Among the samples subjected to different pretreatments, US30 and BL10 had the smallest size (*p* < 0.05) (Figure 2). It has been reported that prolonged heating effectively reduces the number and length of the calcium oxalate needle-like crystals in taro flowers and blunts their tips, thereby mitigating the irritating properties of these crystals; specifically, a reduction of approximately 80% in crystal length and approximately 70% in crystal number was observed following 120 min of heating [33]. The processing-induced chemical reactions and alterations in cell membrane permeability may have led to increased oxalate leaching [8]. Crystal lengths also decreased with increasing ultrasound duration (*p* < 0.05). This indicates that the high ultrasonic amplitude and duration within the structure caused the breakdown of raphide crystals/shrinkage in size. The findings in this work supported the observation in a study by Sumari et al. [34] that as the ultrasound duration increased, the size of the cellulose particles decreased. The reduction in particle size was caused by ultrasound-generated cavitation energy, which damaged particles through shock waves, localized pressure changes, and fluctuations. This led to wear and fragmentation, decreasing the particle size [34]. The sizes of the FT and US20 samples showed similar changes (*p* > 0.05). Pretreatment applications generally resulted in smaller crystal sizes compared to drying methods. This indicates that smaller crystals can be obtained with shorter pretreatment times. The results showed that the cells were affected differently by the various treatments, which led to variability in the size of the calcium oxalate crystals. Studies evaluating the relationship between calcium oxalate levels in plants and different treatments are very limited; therefore, further studies are needed to analyze the decreasing mechanism of calcium oxalate with different processes.

## 3. Materials and Methods

### 3.1. Sample Preparation and Drying Procedure

The plant samples were collected from the Yüksekova District of Hakkari, Türkiye, in 2024. The plant samples were identified as *Arum rupicola* Boiss. var. *virescens* (Stapf) P.C. Boyce at the Hakkari University Biodiversity Research Center (Herbarium Code VPH381) by Assoc. Prof. Dr. Muzaffer Mükemre. The samples brought to the laboratory were washed, and the stalks were removed. The leaves were cut by hand into 5 cm lengths and then used in the pretreatment and drying processes.

#### 3.1.1. Pretreatment Applications

Ultrasound (US): The samples were sonicated at 20 kHz and 200 W using a probe (Bandelin, UW 3200, Berlin, Germany) connected to an ultrasonic generator (Bandelin Sonopuls, HD 3200, Berlin, Germany). Ultrasound applications were performed at 100% amplitude for 10, 20, and 30 min. All ultrasonic applications were performed at 32 ± 2 °C, with temperature control maintained using ice packs.

Blanching (BL): The samples were immersed in water at 90 °C for 5 and 10 min.

Frozen (FT): The samples were frozen at −18 °C for 16 h and then thawed at room temperature for one hour.

For all immersion procedures in the pretreatment stage, 15 g of sample and 300 mL of solvent were added to a glass beaker (500 mL). The ratio between the sample and the immersion solution (1:20, *w*:*v*) was determined through preliminary experiments.

#### 3.1.2. Drying Processes

Arum samples were dried by infrared, microwave, conventional, shade, and sun drying methods. A novel double-sided infrared dryer (Uniterm, Ankara, Turkey) was combined with a DOA-P730-BN vacuum pump (Gast Manufacturing, Benton Harbor, MI, USA) for infrared drying. The drying system consists of four 250 W infrared lamps placed both on the ceiling and floor inside the oven. The distance between the samples and the lamps was maintained at 12 cm. The samples were dried at 250 W infrared power under 200 mmHg vacuum pressure. Microwave drying (MS23J5133AT/TR, Samsung Electronics, Klang, Malaysia) was performed at 300 W. Conventional drying (Memmert, UN 55, Schwabach, Germany) was performed at 70 °C. For shade (18 °C ± 2) and sun drying (25 °C ± 2), the samples were spread out on a cloth. The drying processes were continued until the moisture content of the samples decreased to 7.5–9.5% (dry basis). The oven-drying method was applied to determine the moisture content of the samples [35]. The drying process was performed five times.

### 3.2. Preparation of Methanolic Extracts

Ground fresh (5 g) and dried samples (1 g) were mixed with 15 mL of methanol:water (80:20, *v*/*v*). The mixture was shaken at 150 rpm for 2 h at room temperature on an orbital shaker. The mixtures were centrifuged at 8000× *g* for 10 min, after which the supernatant and residue were separated. A second extraction was performed on the residue using 10 mL of the same solvent. The supernatants were collected and adjusted to a final volume of 25 mL with extraction solvent [36]. The methanolic extracts were used for the analysis of polyphenols and 5-hydroxymethylfurfural (HMF).

### 3.3. Total Phenolic Content (TPC) and Total Flavonoid Content (TFC)

TPC was determined using the Folin–Ciocalteu method, and the results were expressed as mg gallic acid equivalent (GAE)/kg DM [37]. TFC was determined according to Zhishen et al. with the results expressed as mg catechin (CE)/kg DM [38].

### 3.4. Antioxidant Activities

The DPPH and ABTS (Sigma-Aldrich, St. Louis, MO, USA) assays of the methanolic extracts were carried out following the methods of Pyo et al. [39] and Re et al. [40], respectively. Both results were expressed as Trolox equivalent antioxidant capacity (mmol Trolox equivalent/g DM).

### 3.5. Phenolic Compounds and HMF

The individual phenolic content and HMF were simultaneously determined with an HPLC system (Shimadzu, Kyoto, Japan). The HPLC system was equipped with an LC-20 AD pump, CTO-10AS VP column oven, and SPD-M20A diode array detector. The determinations of phenolics and HMF were carried out according to Colaric et al. with minor modifications [41]. A Symmetry C18 column (250 × 4.6 mm, 5 μm; Waters, Milford, MA, USA) at 25 °C was used for separation. The mobile phases consisted of 2% acetic acid in water (A) and 0.5% acetic acid in water:acetonitrile (1:1, *v*/*v*) (B). The flow rate was set at 1.0 mL/min. The gradient program was as follows: min 0, A (90%)–B (10%); min 30, A (80%)–B (20%); min 60, A (65%)–B (35%). HMF was detected at 276 nm, and phenolic acids were detected at 320 nm. Quantification of phenolics and HMF (Carl Roth, Karlsruhe, Germany) was performed using their respective standards.

### 3.6. Total Chlorophyll Content Determination

The total chlorophyll content was determined according to the method reported by Arnon [42]. After homogenizing 0.25 g of the sample in 80% acetone, it was centrifuged at 2000 *g* at 4 °C for 15 min. The supernatant (2 mL) was adjusted to 10 mL with 80% acetone. The absorbances were read at 663 and 645 nm. The results were calculated according to Equation (1).

Total chlorophyll = (20.2 × Abs_645_) + (8.02 × Abs_663_) (1)

### 3.7. Total Carotenoid Content Determination

Approximately 1 g of sample was homogenized with 7.5 mL of methanol:water (70:30) and centrifuged at 4000 *g* for 4 min. The supernatant was transferred to a separating funnel, and the residue was mixed with 7.5 mL of acetone:petroleum ether and then centrifuged again. This process was repeated twice. The collected supernatants were saponified with 10% methanolic KOH for 60 min. At the end of the time, petroleum ether and NaCI (20%) were added to the separating funnel, the upper layer was washed with water, Na_2_SO_4_ was added, and the mixture was filtered. The volume of the filtrate was adjusted to 25 mL with petroleum ether. The absorbance was then measured at 444 nm. The results are given as β-carotenoid equivalents and were calculated according to Equation (2) [43].

Total Carotenoid = (Abs × dilution factor)/(extinction coefficient) × 10,000(2)

### 3.8. L-Ascorbic Acid Content

The sample (2 g) was added to 5 mL of 5% metaphosphoric acid and homogenized at 30.000 rpm for 30 s. The homogenate was centrifuged at 8000 *g* and 4 °C for 4 min. The supernatant was passed through a 0.45 µm PVDF syringe tip filter and then injected into an HPLC system connected to an SPD-M20A diode array detector, Shimadzu, Kyoto, Japan. L-Ascorbic acid was separated at 25 °C with a Waters Atlantis^®^ dC18 column (4.6 × 250 mm, 5 µm) (Waters, Milford, MA, USA) using an isocratic elution mode. The mobile phase was water:H_2_SO_4_ (pH: 2.54) at a flow rate of 0.7 mL/min. Measurements were taken at 244 nm. The results are reported as mg/kg DM [44].

### 3.9. Determination of Cyanogenic Glycoside

Distilled water (200 mL) and sample (1 g) were placed in a bottle and allowed to autolyze for 2 h before complete distillation, after which tannic acid was added. The distillate was collected in a conical flask containing 20 mL of 2.5% sodium hydroxide. Then, 100 mL of distillate was mixed with 8 mL of ammonium hydroxide and 2 mL of potassium iodide and titrated with 0.02 M silver nitrate against a dark background to a fixed turbidity endpoint [45,46]. The content of cyanogenic glycoside in the sample was calculated as (Equation (3)).

Cyanogenic glycoside = (Titer Value (cm^3^) × 1.08× exact volume)/(Aliquot volume(cm^3^) × sample weight(g)) × 100 (3)

### 3.10. Preparation of Microscopy Slides

Leaf discs (approximately 3–5) were placed in test tubes containing 30 mL of 5% sodium hydroxide and left overnight. To facilitate the counting of calcium oxalate crystals, the leaf discs were subjected to a cleaning procedure. From a 5% NaOH solution, the samples were treated with commercial household bleach (sodium hypochlorite) until they turned white and then were washed three times in distilled water for at least 10 min per wash. Typically, approximately 30 mL of liquid was used to ensure the samples moved freely. To verify the chemical composition of the crystals, the cleaned samples were treated with 5% acetic acid for 24 h. The samples were washed three times with distilled water for at least 15 min per wash. The clarified samples underwent a dehydration process using 10%, 30%, 50%, 75%, 85%, and 95% reagent-grade ethanol and were finally rinsed with tert-butyl alcohol by shaking for at least 50 min. The samples were placed in xylene for 10 min, with occasional shaking during this time. The clarified samples were placed on glass slides with Canada balsam and examined under a light microscope (Leica DM500, Leica Microsystems, Wetzlar, Germany) using a counter to identify and count the crystals [31,47].

### 3.11. Statistical Analysis

Each analysis was repeated 3 times. The data are presented as the mean ± standard deviation (SD). SPSS 20.0 software (IBM Corp., Armonk, NY, USA) was used for analysis of variance (ANOVA). Differences among the means were determined using Duncan’s multiple range tests (*p* < 0.05).

## 4. Conclusions

In this study, the effects of different drying methods (VCIR, MW, CV, shade, and sun) on certain compounds, including the cyanogenic glycoside content, and calcium oxalate crystals of *Arum rupicola* Boiss. var. *virescens* (Stapf) P.C. Boyce leaf samples were investigated. Additionally, the changes in the cyanogenic glycoside content and calcium oxalate crystal size of fresh samples subjected to different pretreatments (US, BL, and FT) were also determined. All drying methods resulted in decreases in L-ascorbic acid, total chlorophyll, total carotenoid, cyanogenic glycoside content, and raphide crystal size. In addition to these, the TPC, TFC, DPPH, ABTS, and phenolic compounds decreased in samples dried by sun and shade drying. The lowest loss in L-ascorbic acid, total chlorophyll, and total carotenoid contents and the highest TPC, TFC, and antioxidant activity were observed in the sample dried with VCIR. Cyanogenic toxicity and raphide crystals also decreased significantly. Although VCIR is a technology that offers a longer drying time compared to MW, it has been shown to provide a higher quality product in terms of preserving nutrients and antioxidant potential. In particular, the US30 pretreatment or VCIR as a drying method is one of the most effective methods for decreasing the cyanogenic glycoside content and calcium oxalate crystal size. Further studies are needed to determine the effect of different processes and their combinations on the removal of cyanogenic glycosides and, in particular, on the reduction of calcium oxalate crystal size.

## Figures and Tables

**Table 1:** Drying time, moisture, HMF, phenolic compounds, L-ascorbic acid, total chlorophyll, total carotenoid, TPC, TFC, and antioxidant activity of samples.

Sample	Fresh	Vacuum Combined Infrared Drying	Microwave Drying	Convectional Drying	Shade Drying	Sun Drying
Drying Time	-	50 min	10 min	180 min	13 day	8 day
Moisture (%)	88.09 ± 0.15	7.42 ± 0.19 ^a^	8.48 ± 0.20 ^bc^	8.10 ± 0.10 ^b^	9.52 ± 0.22 ^d^	8.71 ± 0.29 ^c^
HMF (mg/kg DM)	-	2.47 ± 0.93 ^a^	16.77 ± 0.50 ^c^	8.58 ± 1.41 ^b^	8.35 ± 0.85 ^b^	17.45 ± 3.49 ^c^
Chlorogenic acid (mg/kg DM)	136.87 ± 5.04	117.28 ± 3.46 ^b^	174.35 ± 5.27 ^c^	81.16 ± 6.40 ^a^	301.75 ± 7.61 ^d^	172.35 ± 16.44 ^c^
Caffeic acid (mg/kg DM)	170.78 ± 37.02	101.06 ± 3.85 ^d^	83.09 ± 1.08 ^b^	93.06 ± 2.35 ^c^	49.62 ± 4.80 ^a^	54.05 ± 1.77 ^a^
L-ascorbic acid (mg/kg DM)	16,209.19 ± 627.04	13,587.74 ± 493.28 ^c^	13,405.77 ± 44.43 ^c^	12,405.76 ± 357.97 ^b^	2808.62 ± 139.79 ^a^	2471.87 ± 230.72 ^a^
Total Chlorophyll (mg/kg DM)	3599.02 ± 107.50	3358.81 ± 39.49 ^e^	3061.19 ± 125.42 ^d^	2038.89 ± 18.98 ^b^	2342.10 ± 119.02 ^c^	1828.88 ± 47.75 ^a^
Total Carotenoid (mg β-car.eq./kg DM)	965.43 ± 60.72	847.90 ± 1.21 ^e^	800.07 ± 15.01 ^d^	627.77 ± 14.14 ^c^	304.83 ± 2.42 ^b^	267.84 ± 2.87 ^a^
TPC (mg GAE/kg DM)	3517.33 ± 69.38	8863.74 ± 43.31 ^e^	7095.83 ± 169.16 ^d^	5215.59 ± 158.23 ^c^	2940.43 ± 76.34 ^b^	2473.86 ± 60.49 ^a^
TFC (mg CE/kg DM)	3220.94 ± 35.21	4029.93 ± 26.47 ^e^	3932.33 ± 65.25 ^d^	3517.47 ± 91.29 ^c^	1588.62 ± 19.03 ^b^	922.63 ± 16.95 ^a^
DPPH (mmol Trolox eq./g DM)	46.10 ± 0.11	48.12 ± 0.19 ^e^	46.79 ± 0.12 ^d^	41.24 ± 0.17 ^c^	36.62 ± 0.10 ^b^	30.79 ± 1.39 ^a^
ABTS (mmol Trolox eq./g DM)	71.07 ± 2.52	66.53 ± 0.44 ^d^	65.23 ± 1.11 ^d^	53.06 ± 4.27 ^c^	34.12 ± 1.04 ^b^	28.11 ± 0.70 ^a^

Data are expressed as mean ± SD. Different lowercase letters indicate significant differences between all values in the same row (*p* < 0.05).

## Data Availability

The data that support the findings of this study are available from the corresponding author upon reasonable request.
